# Surgical Treatment of Propriospinal Myoclonus: A Case Report

**DOI:** 10.7759/cureus.24445

**Published:** 2022-04-24

**Authors:** David L Baker, Thomas J Harkey, Mohammed H Khaleel, Antonio T Howard, Viktoras Palys

**Affiliations:** 1 Department of Neurosurgery, University of Arkansas for Medical Sciences, Little Rock, USA; 2 Department of Physical Medicine and Rehabilitation, University of Arkansas for Medical Sciences, Little Rock, USA

**Keywords:** surgical treatment, segmental myoclonus, treatment choices, long-term outcome, propriospinal myoclonus

## Abstract

Propriospinal myoclonus (PSM) is a rare segmental movement disorder characterized by repetitive irregular myoclonic jerks of the trunk and/or axial limbs at the resting state. It is imperative to make a correct diagnosis as other movement disorders can be mistaken for or mask PSM. Therefore, a battery of neuroimaging and neurophysiological testing must be undertaken. In our case report, we discuss a patient who was diagnosed with PSM concurrently with cervical degenerative stenosis and then had a successful outcome via surgical decompression and arthrodesis of the cervical spine. We documented the patient’s postoperative course and achievement of complete remission, sustained at a 41-month follow-up. We then grouped our case together with five other PSM cases in the literature to offer readers a broader context of the role of surgical spinal intervention in ameliorating PSM.

## Introduction

Propriospinal myoclonus (PSM) is a rare disorder characterized by repetitive, irregular, myoclonic jerks that classically start in the midthoracic spinal segments and slowly propagate to adjacent segments via the propriospinal pathways, resulting in flexion or extension of the trunk, neck, knees, and hips [1]. Segmental myoclonic disorders of spinal origin have been documented for some time [2], but propriospinal myoclonus was first differentiated in 1991, due to its peculiar localization and spreading pattern. ‘Propriospinal’ in this case refers to neurons that originate and have axons that terminate within the spinal cord, and which help coordinate motor activity between spinal segments [3], in contrast to ‘supraspinal’ projections such as corticospinal, rubrospinal, and vestibulospinal tracts. The number of propriospinal neurons in spinal cord gray matter is believed to be greater than the number of motoneurons [4]. Propriospinal fibers form fasciculi proprii in the innermost layer of the white matter of the spinal cord [3], which are noted to have a relatively slow conduction velocity when compared to other spinal axons. This difference allows for the selective detection of activity in propriospinal axons in the workup for PSM. Although, there is data to suggest that these axons can be voluntarily activated by normal individuals mimicking PSM [5].

PSM cases in the literature are diverse in presumed etiology and in clinical features. Van der Salm’s 2014 study chose to divide them into primary (idiopathic), secondary (infection, toxin, lesion, or trauma), and functional categories [5]. The same study claimed that secondary cases due to structural lesions are relatively rare; only 12 out of 179 reported PSM cases (7%) were due to ‘structural spinal cord lesions’ [5]. Ischemic myelopathy, cervical tumors, neuromyelitis optica, syringomyelia, cervical disc herniation, and trauma to the back are examples of other spinal cord lesions that could potentially trigger PSM [5]. Possible infectious triggers include herpes zoster virus, hepatitis C virus, Lyme neuroborreliosis, and in two cases *E. coli* toxin [5]. There were also a handful of cases in which medications (such as interferon, ciprofloxacin, and bupivacaine) apparently activated PSM [5]. There is a solitary case report from India of a patient who developed PSM after spinal surgery [6]. A large percentage of cases reported have been deemed “functional” (psychogenic) in origin, e.g., 104 out of 179 (58%) of those identified by van der Salm et al. [5].

Diagnosing myoclonus of organic spinal origin involves eliminating other potential causes, including jerks of cerebral origin (e.g. epileptiform discharges) and compulsive, functional jerks (e.g. tics), as well as with certain drugs and Lewy-Body disorders [1,7]. A variety of clinical features are typically associated with PSM, though their utility in differentiating functional and organic causes is limited [5]. One commonly noted clinical feature of PSM is that lying supine, or possibly the sleep-wake transition period, can elicit or exacerbate myoclonic activity, especially in those cases of idiopathic rather than functional origin [8]. “Distractability”, the ability of an alternate, mind-occupying task to reduce symptoms would suggest functional origin to those symptoms, many cases of physiological origin were noted to have distractible symptoms as well. Therefore, distractibility is not a very specific finding for functional PSM [5].

Following a suggestive clinical presentation, a battery of neuroimaging and neurophysiological testing is undertaken. Magnetic resonance (MR) imaging of the neuraxis helps to diagnose concurrent conditions and potentially triggering lesions. Electrophysiological studies, such as electromyography (EMG) and electroencephalography (EEG) are also used to appreciate burst patterns and rule out epileptiform activity, respectively. Polymyography, which uses surface electrodes to record from multiple muscles, is often used to measure nerve conduction velocities and EMG burst durations and can be helpful in identifying the characteristic propagation of jerks to adjacent muscle groups.

## Case presentation

Our patient is a 70-year-old right-handed Caucasian white female who has provided written consent for this publication. The patient presented with seven years of myoclonic truncal jerking activity described as the sudden contraction of abdominal muscles. Symptoms started abruptly and were gradually worsening in intensity, frequency, and duration. Eventually, symptoms began occurring throughout the day, primarily while she was at rest, and were worst in the supine position. However, when the patient was engaged in certain activities of daily living, especially those requiring concentration (e.g., quilting), the jerking movements were decreased in frequency and severity. The patient denied any premonitory urge to move or unpleasant sensation relieved by movements. She also reported no abnormal sensation or movement in her legs. The patient was treated with pramipexole with some success but did not respond to levodopa-carbidopa, clonazepam, or carbamazepine trials. Over the eight months preceding her surgery the patient noticed neck pain along with radiculopathic and myelopathic symptoms, including tingling sensation and pain radiating to right I-III digits, gait clumsiness, and dropping objects from her hands. The patient was also diagnosed with diabetic neuropathy, chronic kidney disease, and Sjögren’s syndrome.

The neurological examination was notable for flit-like brief myoclonic jerks occurring every several seconds predominantly in abdominal musculature with occasional concomitant hip flexion. These movements were distractible, though a few jerks were still seen during ambulation and tasks requiring mental concentration. The jerks intensified in the supine position. The patient did not have myelopathic long tract signs on physical examination, which could have been masked by neuropathy, and was intact on motor and sensory examination.

**Figure 1:**
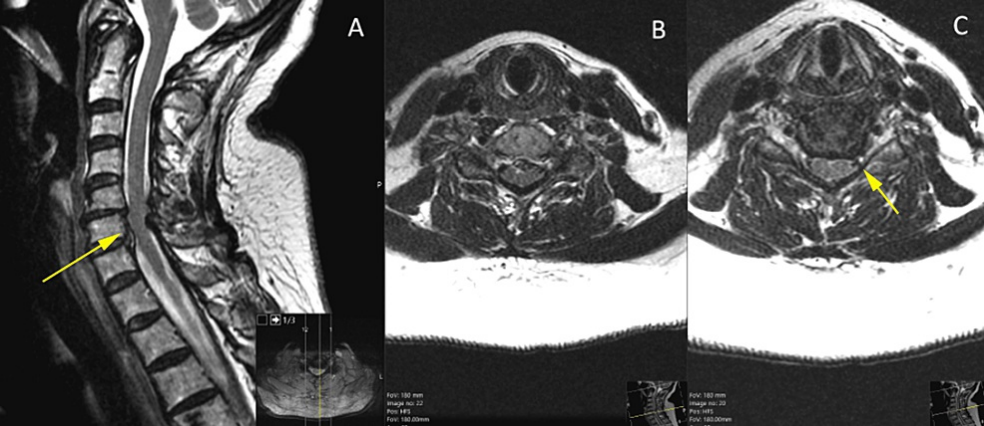
Pre-operative MRI-T2 image Pre-operative MRI-T2 demonstrating central spinal stenosis (anterior-posterior diameter 6 mm) at C5-7 resulting from disc-osteophyte complexes and ligamentum flavum hypertrophy. A. Sagittal view: yellow arrow indicates the area of maximal compression; B. Axial view at the level of maximal cord compression; and C. Axial view: yellow arrow indicates left neuroforaminal stenosis at C5-6.

Diagnostic imaging included an MRI of the brain, which was unremarkable except for a small focus of hemosiderin in the left parietal white matter. MRI of the cervicothoracic spine showed cervical spondylosis causing central canal stenosis, with an anterior-posterior diameter of 6 mm at the C6 level, as well as left C5-6 and right C6-7 foraminal stenosis. MRI showed no signal T2 signal change in the spinal cord (Figure 1).

Electromyography (EMG) findings were obtained in the supine, seated, and standing positions. In the seated position, the EMG surface discharges were described as frequent and arrhythmic, 100-150 millisecond (ms) bursts in the proximal lower extremity muscles. In the supine position, they were described as 50-100 ms bursts, beginning in the abdominal muscles and quickly followed by proximal lower extremity muscles, with co-contraction of agonist and antagonist muscle groups. Waxing and waning tonic surface EMG activity were also noted in the thoracic paraspinal muscles, consistent with poor relaxation. When standing, frequent arrhythmic discharges of 50-100 ms duration, followed by lumbar, paraspinal, trapezius, and proximal, then distal leg muscles, with a co-contracting pattern of muscle activation. Similar surface EMG changes were seen with goal-directed movements, but these were markedly less frequent. All of the above-described EMG surface discharges were associated with jerking movements of the trunk.

Electroencephalography was also performed, but no activity corresponding to the aforementioned EMG changes was found. Laboratory studies were unremarkable, except for a low thyroid-stimulating hormone (TSH) level and a positive antinuclear antibodies (ANA) test (expected in Type 1 Diabetes).

Given the cervical spine pathology in the MRI study, the patient was referred for neurosurgical evaluation. A thorough discussion of the risks and benefits of surgical decompression of the cervical spinal cord took place, with emphasis on the uncertainty of predicting surgical benefit in PSM. The patient continued to show interest in surgical treatment. Subsequently, C5-C7 laminectomy, non-instrumented arthrodesis along the posterior lateral mass surfaces using morselized autograft, and C5-C7 anterior cervical discectomy and fusion using anterior plate were performed in one stage with polyetheretherketone (PEEK) interbody cages filled with morselized bone autografts used for disc spaces. Postoperatively, the patient wore a cervical collar.

At her six-week postsurgical follow-up, the patient reported 99% resolution of PSM symptoms and full recovery of right arm sensory changes. A hard Aspen cervical collar was recommended for 10 weeks post-operatively, due to a history of delayed fracture healing. At the six-month postsurgical follow-up, the patient reported being symptom-free and did not report any neck pain. On her 18-month follow-up, however, she said the clonus had returned shortly before the visit. It only occurred when lying down, and the jerks resolved with standing or applying the cervical collar.

At the 21-month follow-up, these jerks had worsened significantly. The jerks were diminished but did not resolve entirely with the cervical collar. These symptoms were accompanied by intermittent tingling of the hands, as well as a new difficulty swallowing, which preceded the jerk recurrence. A swallow study revealed mild narrowing near the level of the laminectomy and fusion. The dysphagia had progressed to the point she required esophageal dilatation twice, though neither helped her symptoms.

**Figure 2:**
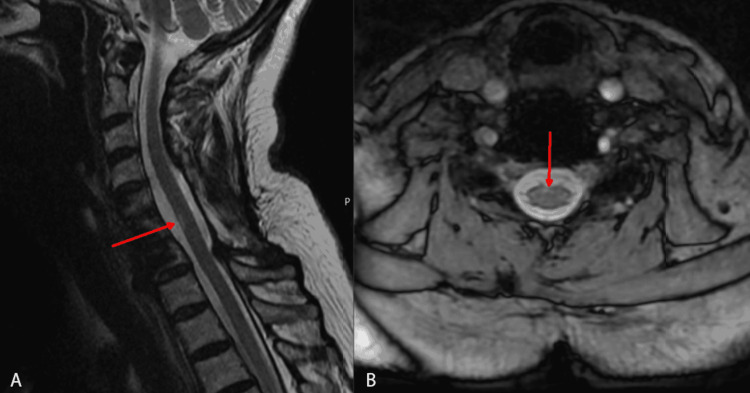
MRI-T2 image at 21 months post-operatively. A: Sagittal view, the red arrow indicating the small focus of myelomalacia along the anterior median sulcus of the cord at the C6 level; B: Axial view, red arrow again indicating myelomalacia of cord at C6.

At this point, a cervical spine MRI was obtained and demonstrated adequate spinal cord decompression with a circumferential CSF cuff. However, a small (6 x 1 x 1 mm) focus of new myelomalacia along the anterior median sulcus of the cord at C6 was visible (Figure 2). To address her symptoms, one-week trials of both baclofen and diazepam were attempted, but both were unsuccessful.

After 24 months of the operation, the patient claimed there were now spaces of several nights in which she had no jerking symptoms, followed by one or two nights of symptoms. Her dysphagia persisted, and she noted bilateral hand numbness.

**Figure 3:**
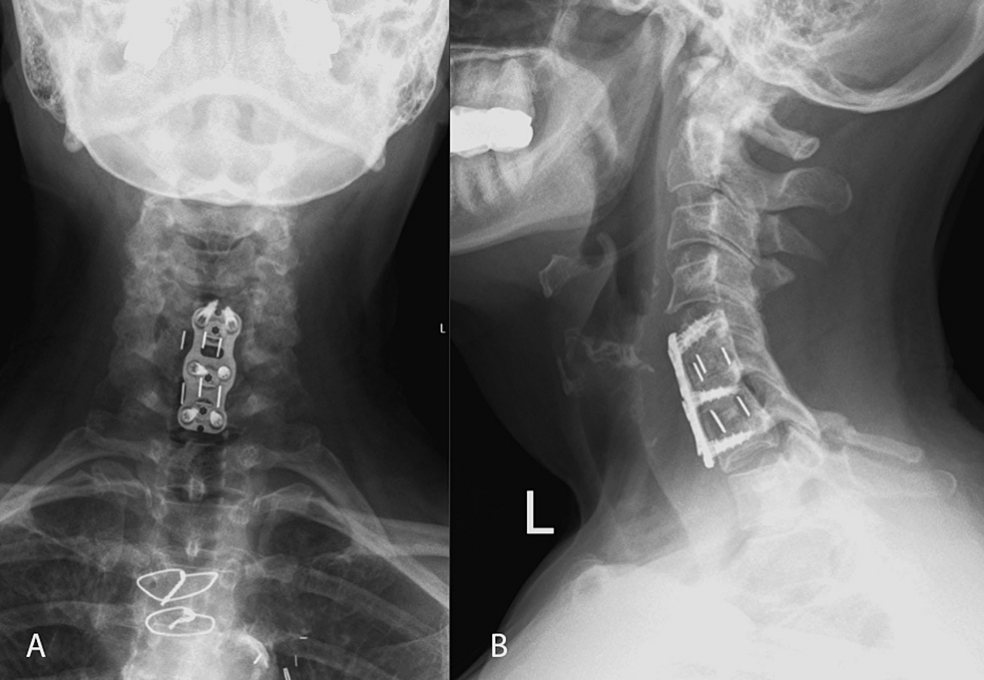
X-ray following C5-7 laminectomy and anterior cervical discectomy and fusion. An X-ray was taken 28 months post-operatively that demonstrated expected postsurgical changes with no complications. A: Coronal view; B: Sagittal view.

After 27 months of surgery, the jerks had almost completely resolved, and only manifested when she became tense or cold. At this point, she had ceased taking muscle-relaxant medications. She was primarily bothered by the bilateral persistent hand numbness on the palmar surface (with sparing of the dorsal surface). These symptoms were also associated with burning pain in the palmar surface of hands and fingers as well as in the lower legs, and they were identified as consistent with diabetic neuropathy. Plain radiographs demonstrated expected postsurgical change with no complications, along with chronic C7 spinous process fracture (Figure 3).

**Figure 4:**
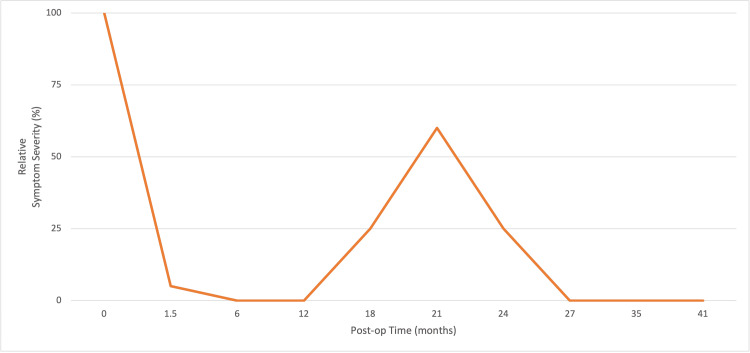
Postoperative symptom severity versus time. The X-axis represents the time since surgery (in months). The Y-axis represents the relative severity of jerking symptoms, with 100% being the baseline immediately before surgery.

After 35 months of surgery, the patient reported being asymptomatic with complete remission of myoclonic jerks over the past several months along with the resolution of burning hand and lower leg paresthesias. The latter coincided with the start of duloxetine. The patient reported that this remission of symptoms was sustained at the most recent check, 41 months post-operatively. The clinical trajectory in the postoperative period is depicted in Figure 4 below. 

## Discussion

PSM has been notoriously unresponsive to conservative treatment. Some PSM cases had symptomatic success with clonazepam, botulinum toxin, baclofen, and anticonvulsants like valproate, though the unclear etiology of PSM makes it difficult to explain this variable response to medical therapy [5,8].

Due to the rarity of PSM, there are few reports of surgical treatment, and there are no guidelines for surgical management of PSM. Though there are many instances in the literature of myoclonus of spinal origin being treated successfully with surgery [9-11], our review yielded only five reports of favorable outcomes for a PSM patient after the surgical treatment of spine pathology [12-16]. We have included these cases along with our case in Table 1 below.

**Table 1:** Case reports describing surgical management of Propriospinal Myoclonus. The "Follow-up" column reports the latest documented follow-up with the patient. The "Surgical treatment" column varies in detail due to varying levels of description supplied in case reports. Capelle et al. [9]; Shprecher et al. [10]; Jang et al. [11]; Marrero-Gonzalez et al. [12]; Tapia Perez et al. [13]

Publication year	Authors	Spinal abnormality	Myelopathy	Surgical treatment	Outcome	Follow-up
2005	Capelle et al.	C6 stenosis and nerve root compression	No	Discectomy and decompression with cage placement	Complete resolution of symptoms	1 year
2010	Shprecher et al.	C5-6 disc herniation and nerve root compression	No	Anterior cervical discectomy and fusion	Substantial improvement with some residual symptoms	2 months
2012	Jang et al.	T8-9 disc herniation	No	Discectomy	Substantial improvement with some residual symptoms	5 years
2018	Marrero-Gonzalez et al.	Chiari malformation with C1-2 compression	Yes	Craniectomy and laminectomy	Complete resolution of symptoms	"A few days"
2020	Tapia Perez et al.	C5-6 discectomy complicated post-operatively by C6-7 disc prolapse	Yes	Ventral fusion with plating, followed by second stage decompression	Complete resolution of symptoms	24 months
2021	Our case	C5-7 cervical stenosis	No	Laminectomy, anterior discectomy, and interbody fusion with plate	Complete resolution of symptoms	32 months

The first case of surgical management of PSM was published in 2005. The report describes a patient with C6 nerve root compression due to cervical disc herniation and no cervical myelopathy. The neurosurgeon opted for discectomy decompression of the affected nerve root, followed by cage placement. After the surgery, the patient’s PSM symptoms were significantly improved [12]. In 2010, another case report described PSM symptoms with C5-6 compression due to disc herniation. This compression was not associated with myelopathy, and the patient experienced a substantial reduction in symptoms following surgical decompression [13]. Another report in 2012 described T8-9 disc herniation without myelopathy, in which case, again, the symptoms almost completely resolved after decompression [14]. In 2018, a pediatric case of PSM symptoms involving an Arnold-Chiari malformation causing C1-2 compression with myelopathy was also treated successfully with surgical decompression [15]. A case was reported in 2020 in which an adult patient with C5-6 cervical stenosis with myelopathy developed PSM symptoms one year after C5-6 discectomy. This patient was found to have C6-7 disc prolapse, which was then treated with C5-7 ventral fusion and plating. The symptoms persisted despite no apparent compression and were refractory to medical treatment, so microsurgical dorsal decompressive surgery at C5 with spinal cord stimulator placement was performed. This treatment resulted in a resolution of the patient’s symptoms, but it did require a follow-up procedure when the spinal electrode became displaced from its original site [16]. 

Despite these scattered cases, no clear set guidelines exist for the management of PSM arising from organic causes, and most clinicians opt to maximize conservative measures in the attempt to suppress myoclonic activity. We hope that the successful postoperative outcome in our patient contributes to the current literature and refreshes the collective knowledge of PSM. Of particular note, our case has three years of follow-up, showing a temporal course of clinical response of PSM symptoms with a remission, exacerbation, and eventual remission as likely a durable response.

It is debatable whether circumferential (posterior-anterior) spinal cord decompression and/or arthrodesis were necessary for our patient. However, due to the overall uncertainty of the surgical role in PSM, we chose to radically eliminate both static and dynamic components of spinal cord irritation. The complete resolution of PSM symptoms supports our preoperative hypothesis that spinal cord compression may have been contributory to her symptoms. Distractibility was a problematic feature in this case, as it might support an argument that our patient’s jerks were possibly functional in origin, despite being a non-specific finding. The durable improvement in symptoms over three years of follow-up and the focus of cervical myelomalacia identified on postoperative MRI make the placebo effect less likely, although it cannot be entirely ruled out in this case. Other events associated with surgery, for example, general anesthesia, should be considered as potential factors in the resolution of the patient’s symptoms. Additionally, cervical stenosis is common, and PSM is rare. It remains to be explained why, if the two are related, the relative frequencies differ so widely.

There is also the question of publication bias for the surgical cases we have identified, as there may be cases in the literature in which PSM was identified along with an operative spinal pathology, and correction did not result in the improvement of symptoms. Unfortunately, the authors could not find these cases in the literature currently, but we would welcome their additions and their inevitable contributions to understanding this pathology.

On a separate note, the location of C6 myelomalacia focus near the anterior median sulcus, where medial vestibulospinal and reticulospinal tracts take the course along with fasciculi proprii, may have a pathophysiological explanation of etiology and clinical course. The theories behind spinal myoclonus include hyperexcitability of local anterior horn cells, including in response to tactile stimuli. Thus, the loss of inhibition from damaged suprasegmental descending tracts (including reticulospinal) may have a pathophysiological role. The time course of over two years before the more durable remission suggests that the damage was likely more of a conduction block (demyelination secondary to external cord compression) type as opposed to a physical disconnection (axonal loss). On the other hand, the medial vestibulospinal tract fibers usually exert a facilitating influence on segmental reflex activity in response to postural changes and tract overactivity may potentiate myoclonus. The relief of dynamic irritation to the medial vestibulospinal tract with surgical arthrodesis may explain the immediate although temporary postoperative symptom improvement and, notably, the association with the positive stabilizing effect of a cervical collar. In other words, it all suggests that there was a dynamic component of spinal cord irritation, in addition to the static cord compression. Based on our case, we suggest the elimination of both the static and dynamic components of spinal cord microtraumatization when choosing a surgical treatment strategy, with the exact degree of the spinal cord decompression required in PSM remaining to be determined.

## Conclusions

Propriospinal myoclonus has historically been largely managed medically, but rarely successfully. Due to the heterogeneity of the etiology of PSM, there are no established guidelines for the management of PSM. Our patient presented with myoclonus which was noted to be distractable in nature, who was poorly responsive to medical therapy, and who had concomitant radiculopathic and myelopathic symptoms. She received a diagnosis of PSM based on EMG and clinical findings and was found to have canal and foraminal stenosis in the cervical spine on MRI. Following a C5-7 laminectomy, discectomy, and fusion, the patient initially improved though with some symptom recurrence, worst at the 21-month post-operative mark, then improving again to complete resolution sustained at 41 months post-operatively, and is currently taking only duloxetine for medical management. Our case, together with the five discussed above, suggests that surgery can be a viable means of treatment for PSM in cases where it is indicated by the pathology. Furthermore, the long-term success associated with our case indicates that a surgeon’s preoperative discussion with a PSM patient may include a real hope of symptom remission.
